# Network localization of altered auditory and somatosensory sensitivity based on causal brain lesions

**DOI:** 10.1093/braincomms/fcaf463

**Published:** 2025-11-24

**Authors:** Shreya Tripathy, Gillian N Miller, Alexander L Cohen

**Affiliations:** Department of Neurology, Boston Children’s Hospital, Harvard Medical School, Boston, MA 02115, USA; Boston University Chobanian and Avedisian School of Medicine, Boston, MA 02118, USA; Department of Neurology, Boston Children’s Hospital, Harvard Medical School, Boston, MA 02115, USA; Department of Neurology, Boston Children’s Hospital, Harvard Medical School, Boston, MA 02115, USA; Center for Brain Circuit Therapeutics, Brigham and Women's Hospital, Boston, MA 02115, USA; Department of Neurology, Harvard Medical School, Boston, MA 02115, USA

**Keywords:** lesion network mapping, functional connectivity, sensory processing, stroke, brain injury

## Abstract

Sensory processing as a neurological construct is the perception and interpretation of sensory information from both the body and the environment. Disruptions to sensory processing adversely impact daily functioning. One type of disruption that is particularly interesting is altered sensory modulation, leading to hypersensitivity or hyposensitivity, which are common in many neurodevelopmental and psychiatric conditions such as autism spectrum disorder, attention-deficit/hyperactivity disorder, Alzheimer’s, and schizophrenia. Here we aim to identify modality-specific and cross-modality brain networks involved in altered auditory and somatosensory processing. A systematic review identified 61 patients with new-onset sensory alterations following focal brain injury. Lesions were traced and combined with resting-state data from 1000 healthy controls to generate normative lesion connectivity maps. The specificity of our cohort’s lesion-connectivity compared to lesions associated with 22 other neuropsychiatric symptoms was assessed with voxel-wise two-sample *t*-tests performed with the FSL Permutation Analysis of Linear Models tool (family-wise error *P* < 0.05). A conjunction analysis against lesions associated with hallucination was conducted by binarizing and multiplying one-sample T-test maps to identify common lesion-connections between the conditions. Modality-specific networks were connected to their unimodal cortices and the cerebellum. Overall, lesions associated with cross-modality sensitivity changes had convergent connections to the substantia nigra, medial orbitofrontal cortex, and cerebellum (Lobule V, medial Lobule VIIIa). Subgroup analysis by direction revealed that lesions causing decreased sensitivity were connected to lobule X, in addition to the aforementioned cerebellar regions, while those causing increased sensitivity were only connected to medial V and bilateral V. Regardless of directionality, 90% of lesions exhibited connections to bilateral Lobule V. Conjunction analysis with hallucinations revealed common lesion-connections to cerebellar vermis and frontal pole. Our analysis identified significant lesion-connections to the substantia nigra, medial orbitofrontal cortex, and cerebellum—highlighting these key regions in cross-modality sensory processing. These findings emphasize the role of higher cognitive functions in sensory integration and suggest potential targets for neuromodulation to improve sensory processing.

## Teaser

Lesions causing altered sensory intensity are consistently connected to the substantia nigra, medial orbitofrontal cortex, and cerebellum− highlighting these regions in cross-modality sensory processing. Interestingly, the cerebellar vermis is a convergence point between lesions causing altered sensory intensity and hallucinations, indicating the regions involvement in clinically relevant sensory experience alterations.

## Graphical Abstract

**Graphical Abstract:**
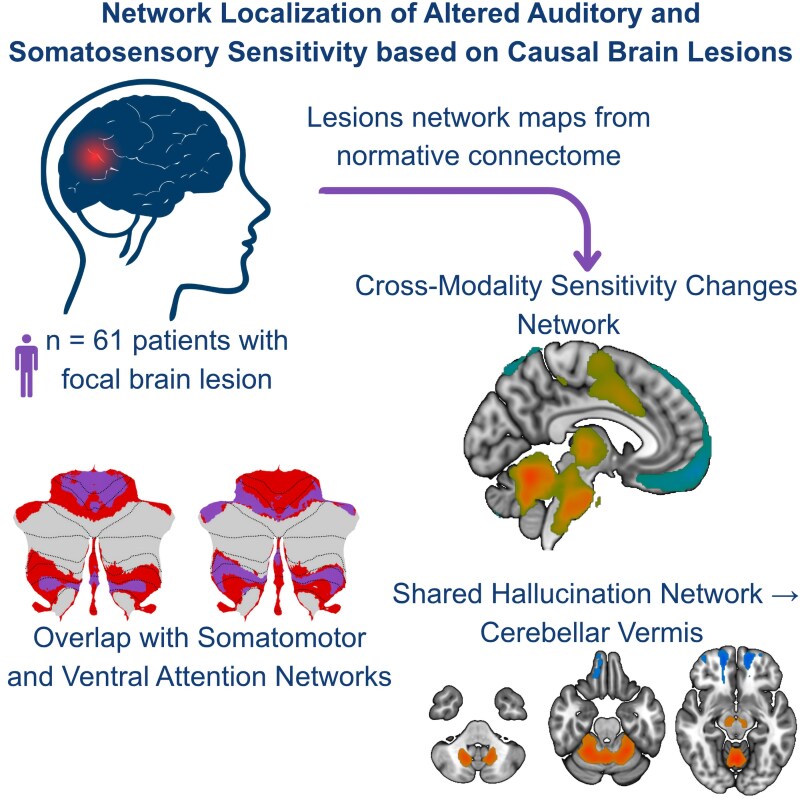

## Introduction

Sensory processing as a neuropsychological construct is the perception and interpretation of sensory information from both the body and the environment. Disruptions to sensory processing adversely impact daily functioning, psychological well-being, and quality of life.^1-3^ While brain injuries can sometimes lead to complete absence of a sensation, i.e. blindness, deafness or anaesthesia due to disruption of primary sensory pathways, one type of disruption that is particularly interesting is altered sensory modulation, leading to hypersensitivity or hyposensitivity, as opposed to complete absence of sensation or even insertion of an exogenous sensation/pain. Alterations to sensory processing or sensitivity are common in many (non-lesional) neurodevelopmental and psychiatric conditions such as autism spectrum disorder (ASD), attention-deficit/hyperactivity disorder (ADHD), Alzheimer’s, and schizophrenia,^4,5^ yet the neuroanatomical basis for these alterations remains a matter of debate. Thus, new approaches for identifying consistent structures involved in altered sensory processing may improve understanding of a wide variety of conditions and potentially highlight new therapeutic approaches to avoid serious injury.

The majority of existing neuroimaging studies have typically assessed sensory processing through the lens of a single domain at a time. For instance, somatosensory impairments have been localized to several regions, including somatosensory cortex I (SI), somatosensory cortex II (SII), thalamus, prefrontal, and insular cortex.^6-9^ Similarly, altered auditory processing has been linked to primary auditory cortical regions, the amygdala, and the insular cortex, among others.^10-12^ Furthermore, the vast majority of this work utilizes neuroimaging correlates, i.e. group-level fMRI or PET activations or deactivations, and it remains unclear which identified brain regions are a *driver of* the sensory alteration and which are *compensating for* the sensory alteration. In other words, the question remains: ‘which brain regions, when directly altered by injury or neuromodulation, drive changes in sensory modulation?’ A recent methodological advance is ‘lesion network mapping’, which identifies specific brain networks, rather than single regions, causally involved in a symptom by integrating lesion cohorts associated with new-onset symptoms and high-quality functional connectome maps representing typical brain network organization.^13,14^ Lesion network mapping has had success in localizing domain-independent networks for some sensory phenomena, such as visual and auditory hallucination and visual and motor anosognosia.^15,16^ Since hallucinations can be conceptualized as abnormal sensory experiences due to altered sensory sensitivity and predictive coding, both hallucinations and altered sensory sensitivity can be viewed as difficulty with integration or conscious recognition of sensory information. As such, lesions causing hallucinations provide a potentially clinically relevant point of comparison for sensory sensitivity lesions.

In this study, we performed lesion network mapping to investigate the brain network(s) involved in the perception of the strength of auditory and somatosensory stimuli. Using a cohort of patients with lesion-associated disruptions to auditory and somatosensory sensitivity reported in the medical literature, we aimed to (1) identify a domain-independent brain network associated with sensory processing disruptions and (2) explore brain networks linked to domain-specific sensory sensitivity.

## Materials and methods

### Literature search

We performed a systematic literature review to identify studies with: (1) brain injury, (2) altered auditory or somatosensory processing, and (3) an image of their lesion sufficient for mapping. Studies with unclear nature or timing of symptom onset, poor or lack of brain imaging, not in English were excluded. We included case reports, comparative studies, and observational studies on humans. We used the following keywords to identify papers in PubMed from 1 January 1995 to 15 July 2024:

(brain) AND (injury OR lesion OR stroke* OR infarct*) AND [(hyperacusis OR tinnitus OR auditory OR hearing OR acoustic OR aural OR hypoacusis OR deaf OR deafness OR ‘sensory processing’) OR (somatosensory OR tactile OR ‘sensory discrimination’ OR pain OR touch OR temperature OR ‘sensory processing’ or hyperalgesia or allodynia or paraesthesia)] AND ((Magnetic Resonance Imaging) OR (CT)) AND NOT (schwannoma) NOT (cerebellopontine angle)

To maximize the identification of all relevant studies, we performed a forward and backward citation analysis and used the similar articles feature of the database. After reviewing full-text articles (ST), images from the gathered data were aligned and traced by hand onto the ‘MNI152 non-linear, 6th generation, asymmetrical’ atlas brain (2 mm resolution) available distributed with the FSL v6.0 software package^17^ using 3DSlicer v5.7.^18^ For patients with a single published image, 2D lesion masks were created in the same plane as each figure, and for those with multiple images, all slices were combined into a single lesion mask (ST, reviewed by AC) (Supplementary Fig. 1). Patient demographic data (age of symptom onset and sex) were collected as well. Since lesion network mapping synthesizes individual case-level imaging rather than aggregate study data, the risk of bias and certainty assessment were not applicable. The review was not registered.

### Lesion network mapping

#### Lesion connectivity maps

Each lesion served as a seed in a resting state functional connectivity analysis using fMRI data from 1000 healthy young adult controls (500M:500F, ages 18–36) from the Genome Superstruct Project (GSP) normative dataset.^19,20^ A ‘normative’ functional connectivity map was generated for each traced lesion by correlating the average preprocessed time course of the lesion site with the time course of every other brain voxel in each of the 1000 control participants from the normative connectome, and then calculating the group average correlation and T-score map for that lesion, creating a map in which each voxel’s value represents its likely/typical connectivity to a lesion prior to that person's injury occurred.

### Statistical analysis

#### Lesion network overlap

To describe the consistency of strong connections across the lesions within our cohort, ‘lesion overlap maps’ were created by thresholding each lesion connectivity T-score map (*T* > + 7 and < −7, equivalent to a voxel-wise family-wise error (FWE) corrected *P* < 10^−6^)^21^ to create a binarized map of regions functionally connected to each patient’s lesion mask. The binarized maps were then overlapped to identify the number of patients with connectivity to each voxel at the selected threshold.

#### Lesion network sensitivity

Since the overlap procedure above is threshold dependent, lesion connectivity maps were then also statistically analysed with a voxel-wise one-sample *t*-test using the FSL Permutation Analysis of Linear Models (PALM) software using 2000 permutations, tail approximation,^22^ and a voxel-wise FWE-corrected *P* < 0.05. Permutation testing's non-parametric nature and voxel-wise analysis’s granularity aided in controlling for false positives that are common to cluster-based methods.^23^ Furthermore, while thresholded lesion network overlap maps have been included for interpretability, the one-sample *t*-test maps shown below represent the primary statistical results of our study.

### Lesion network specificity

We then assessed the specificity of our sensory processing network(s) to networks characterizing 22 other lesion-related neuropsychiatric symptoms with voxel-wise two-sample t-tests using the FSL Permutation Analysis of Linear Models (PALM) software using 2000 permutations, tail approximation,^22^ and a voxel-wise FWE-corrected *P* < 0.05 (Supplementary Table 2). Each lesion-related neuropsychiatric syndrome was treated as an independent group, with equal weights assigned to each group to ensure an unbiased comparison across different conditions despite varying sample sizes. Using the Buckner *et al*. cerebellar parcellation, we identified overlap between the regions with significant connections and canonical functional brain networks.

#### Conjunction analysis

To identify regions that were both sensitive and specific, we performed a conjunction analysis by binarizing and multiplying together our one-sample and two-sample T-test results, thus yielding regions where lesion-connections were both sensitive and specific to sensory changes.

Additionally, given the sensory symptoms associated with hallucinations and the relevance of sensory gating in other neuropsychiatric conditions, we conducted a conjunction analysis to assess overlap between lesions causing hallucinations from Kim *et al*.^15^ and our sensory cohort, aiming to identify converging evidence.

Since some hallucination-causing lesions specifically led to auditory phenomenon, we also performed a subgroup conjunction analysis restricted to auditory-symptom lesions in both cohorts.

### Subgroup analyses

We repeated the analyses described above with subgroups of our cohort, focusing separately on lesions that caused either an increase or decrease in sensitivity. We further focused on lesions associated with auditory or somatosensory symptoms. Of note, we did not identify any patients with auditory increases therefore we assessed specificity of connections to auditory decrease, somatosensory increase, and somatosensory increase.

## Results

### Lesions associated with sensory change vary spatially but map to a network defined by the cerebellum

**Figure 1:**
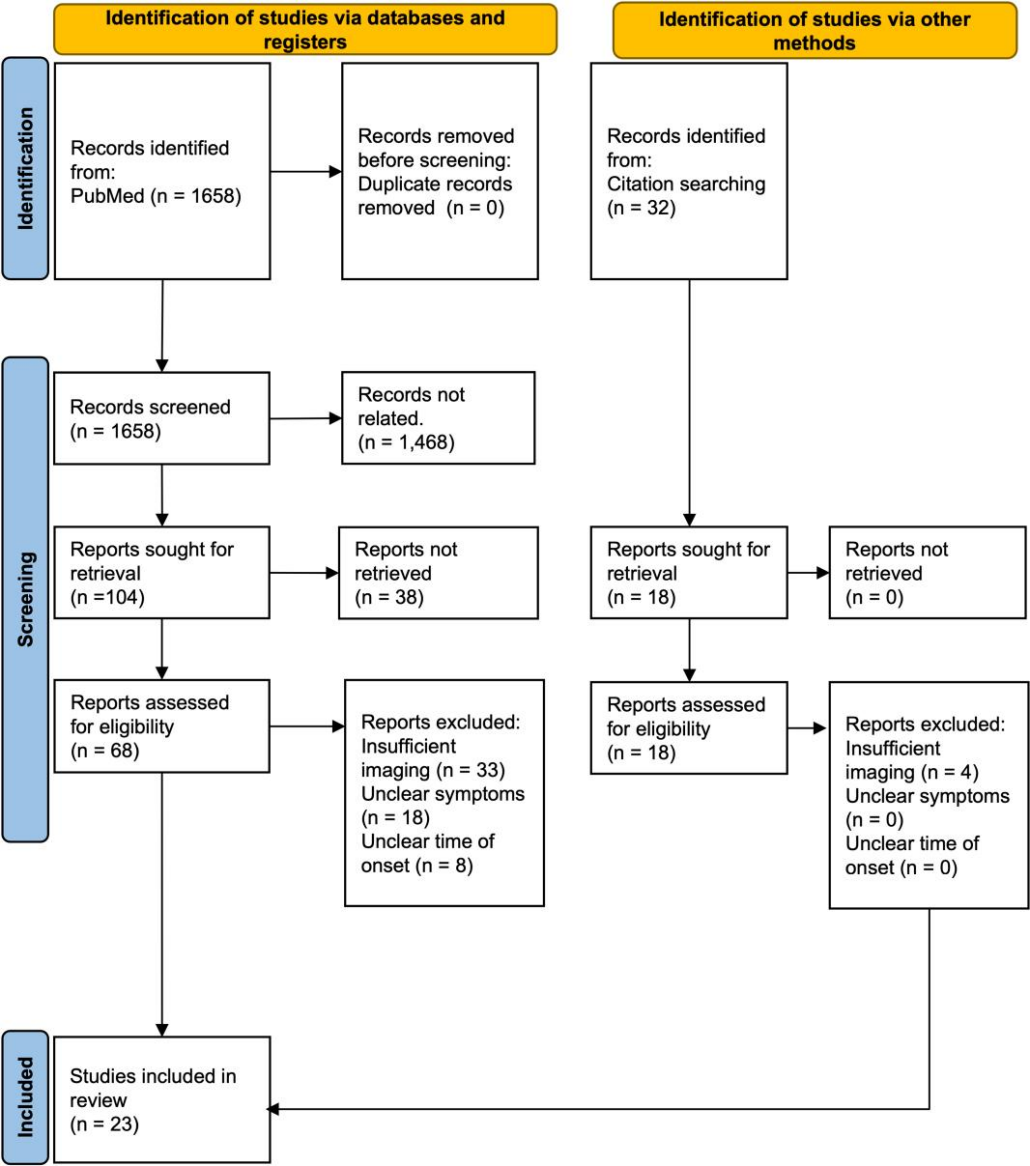
**PRISMA flow chart identifying possible lesions causing altered sensory processing after focal brain injury.** We identified patients with new-onset altered auditory or somatosensory processing following focal lesion with imaging suitable for tracing onto standardized brain atlas via PubMed. A total of 61 patients across 23 studies met these inclusion criteria. Flow diagram adapted from the PRISMA guidelines^24^

The literature search yielded 629 auditory and 944 somatosensory papers. Abstract screening identified 47 auditory and 38 somatosensory studies for full-text review, of which 9 papers representing 12 cases met the inclusion criteria. Forward and backward citation searches and the database’s similar-articles feature added another 14 papers with 49 cases. Overall, 23 studies comprising 61 patients with altered auditory or somatosensory sensitivity after focal brain injury were included (Fig. 1, Supplementary Table 1, Supplementary Fig. 1). The mean age at time of injury was 57.18 years (*SD* = 11.84, 6 patients unknown), with a male predominance (M32:F18, 11 patients unknown). The lesions were spatially heterogeneous with the most common locations thalamus (−25, 17, 12) (*n* = 4), however, this is limited by the use of 2D slice tracings from the literature, which ultimately may miss additional overlap.

**Figure 2:**
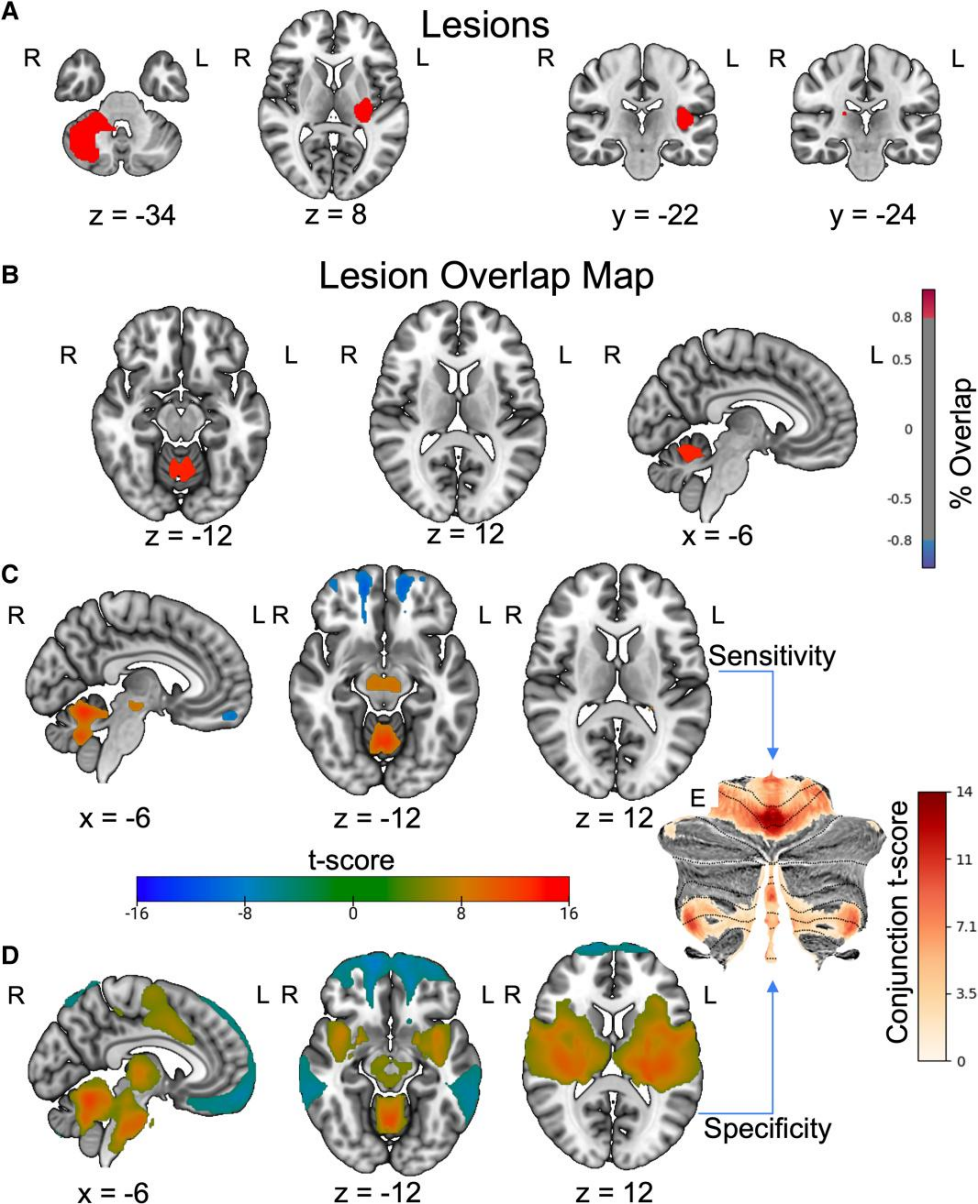
**Lesion network localization of sensory sensitivity**. **(A)** Representative lesions (*n* = 61) causing sensory perception changes, demonstrating heterogeneity of lesion location. **(B)** Lesion overlap map (90%, *T* > 7). **(C)** One-sample *t*-test of lesion connectivity (family-wise error (FWE) corrected *P* < 0.05). **(D)** Functional connectivity specific to lesions causing sensory changes compared to a large cohort of lesions causing other symptoms via two-sample *t*-test (FWE corrected *P* < 0.05). **(E)** Cerebellar regions of interest present in both the one- and two-sample t-tests.

**Figure 3:**
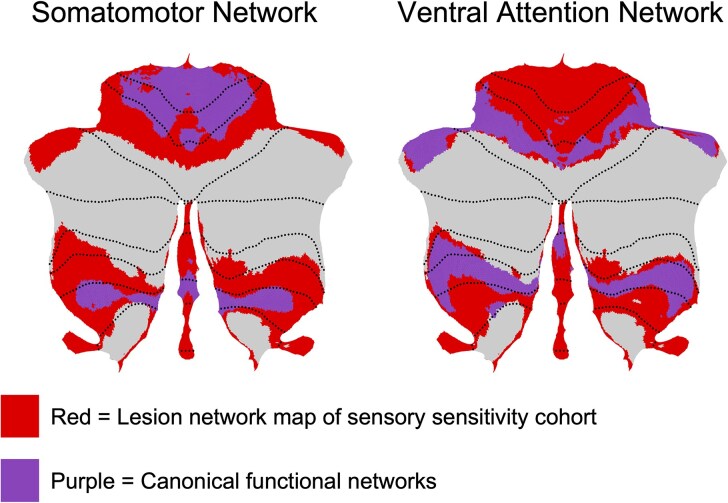
**Key cerebellar regions overlapped with canonical functional networks.** We identified overlap between functional connectivity map of the sensory sensitivity and somatomotor and ventral attention networks, as identified in Buckner *et al*., 2011. The cerebellar map represents lesion network map of the sensory sensitivity cohort and the overlap represents shared regions between our cohort and the canonical functional networks.

However, this limitation is mitigated by using the connectivity profiles of each lesion via lesion network mapping.^25^ We found that 90% (*n* = 55, *T* > 7) of the lesion locations shared positive connectivity with cerebellar medial lobule V (0, −62, −16) and bilateral lobule V (±10, −58, −20) (Fig. 2B). At the chosen threshold, no regions showed consistent negative overlap across patients, indicating that no voxels met this stringent criterion on the negative side. A one-sample *t*-test revealed that these connections were statistically consistent (Fig. 2C). Additionally, there were significant positive connections to cerebellar medial lobule VIIIa (0, −63, −34) and substantia nigra (±6, −20, 12) and significant negative connections to the orbitofrontal cortex (±14, 56, −14) (Fig. 2C). Further analysis revealed that these connections were specific to lesions related to sensory sensitivity when compared to the 659 lesions related to 22 other specific neuropsychiatric symptoms, as were connections to regions of the basal ganglia and insula (Fig. 2D). Using cerebellar parcellation of canonical functional networks, the cohort’s connectivity patterns encompassed the entirety of the cerebellar components of the Somatomotor (SMN) and Ventral Attention Networks (VAN). Specifically, the medial Lobule V and VIIIa corresponded to the SMN and VAN, respectively (Fig. 3).^26^

### Conjunction analysis with hallucinations

**Figure 4:**
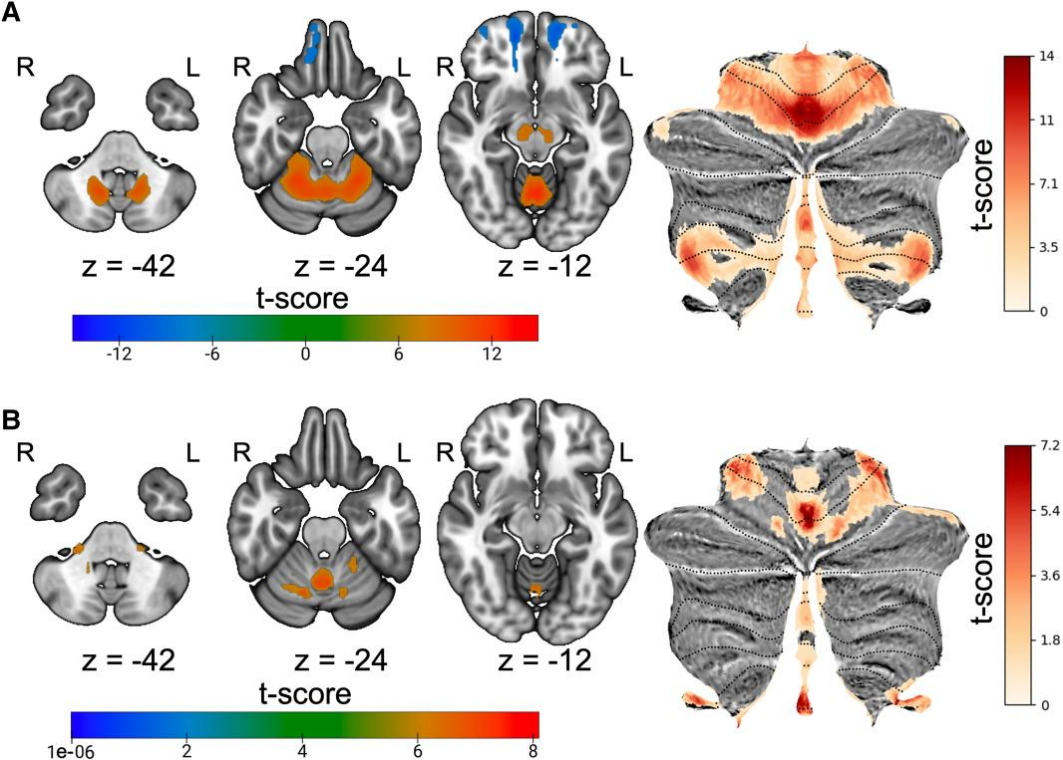
**Conjunction of lesions causing hallucination and sensory changes. (A)** Conjunction of one-sample t-tests of lesion connectivity (family-wise error (FWE) *P* < 0.05) of hallucinations (*n* = 89) and sensory changes (*n* = 61) identified key regions, including the cerebellar vermis. **(B)** Conjunction of one-sample t-tests of lesion connectivity (FWE *P* < 0.05) for lesions causing auditory hallucinations (*n* = 14) and auditory sensory changes (*n* = 14).

Conjunction analysis demonstrated that lesions associated with hallucinations from Kim *et al*. and sensory changes displayed connectivity to the cerebellar vermis and frontal pole (Fig. 4A).

In a subgroup conjunction analysis restricted to auditory-associated lesions, the vermis is again identified as a key node of lesion connectivity (Fig. 4B).

### Independent network connections related to sensitivity increase or decrease

#### Increases versus decreases in sensory changes

**Figure 5:**
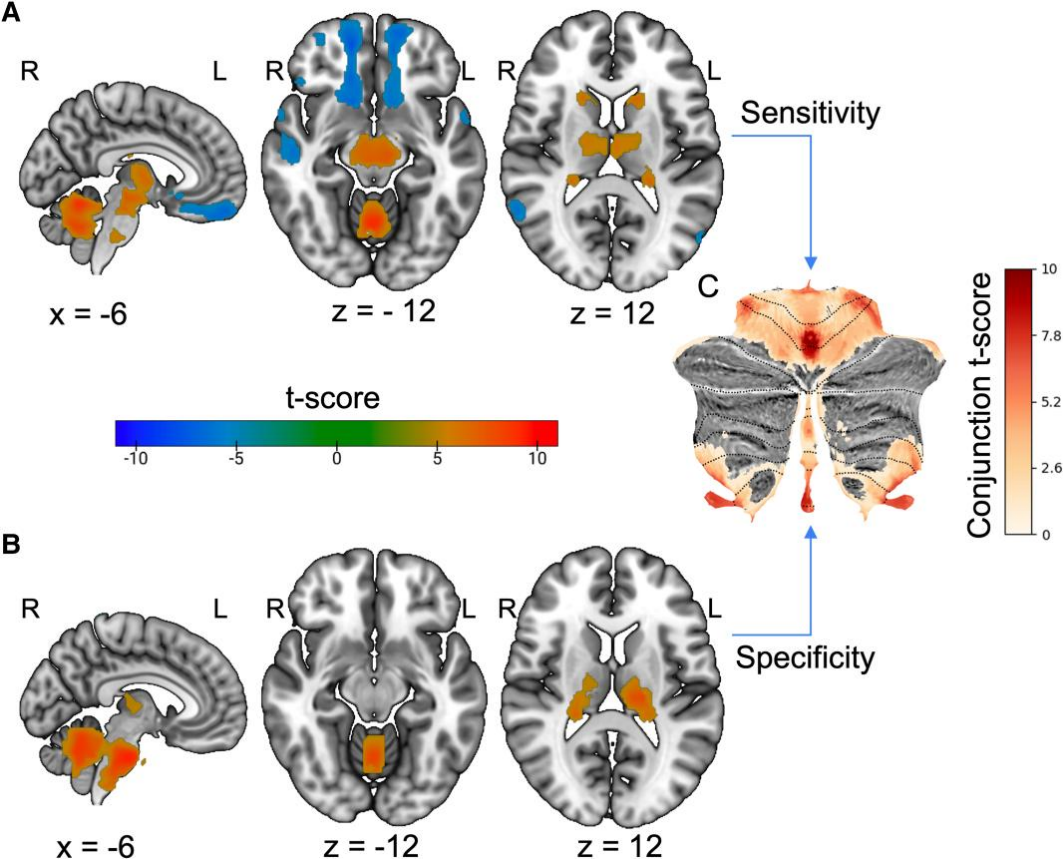
**Lesion network localization of decreased sensory sensitivity. (A)** One sample *t*-test (*n* = 28) of lesion connectivity (family-wise error (FWE) corrected *P* < 0.05). **(B)** Functional connectivity specific to lesions causing decreased sensory changes compared to a large cohort of lesions causing other symptoms via two-sample *t*-test (FWE corrected *P* < 0.05). **(C)** Cerebellar regions of interest present in both the one- and two-sample *t*-tests of decreased sensory sensitivity.

**Figure 6:**
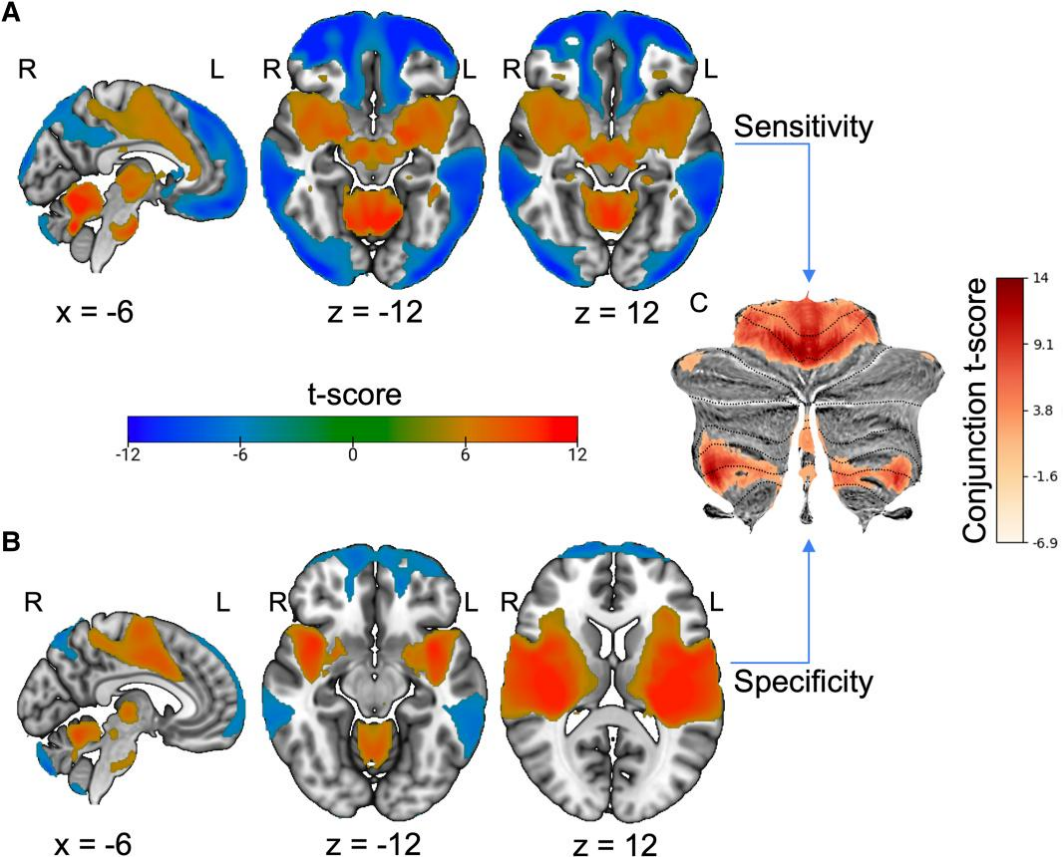
**Lesion network localization of increased sensory sensitivity. (A)** One sample *t*-test (*n* = 33) of lesion connectivity (family-wise error (FWE) corrected *P* < 0.05). (**B)** Functional connectivity specific to lesions causing increased sensory changes compared to a large cohort of lesions causing other symptoms via two-sample *t*-test (FWE corrected *P* < 0.05). **(C)** Cerebellar regions of interest present in both the one- and two-sample t-tests of increased sensory sensitivity.

The cohort was classified by whether the report indicated a decrease or increase in sensory sensitivity (i.e. hypo- and hyper-sensitivity). Of the 28 lesions associated with *decreased* sensitivity, 89% (*n* = 25, *T* > 7) exhibited a positive correlation to medial and bilateral lobule V and medial lobule VIIIa. A one-sample *t*-test found connections to these regions to be statistically consistent, as were connections to the brain stem and Lobule × (Fig. 5A). For the 33 lesions associated with *increased* sensitivity, 90% (*n*  *=* 30, *T* > 7) were positively correlated with medial and bilateral lobule V. A one-sample *t*-test indicated that connections to regions were statistically consistent within the group, as were connections to medial lobule VIIIa, OFC, SN, anterior cingulate, anterior thalamus, and insula (Fig. 6A). The key regions identified in the subgroup one-sample *t*-tests by sensitivity were also specific when compared to 659 lesions causing other symptoms (Table 1).

**Table 1:** Key regions

Regions	MNI	All lesions	Lesions causing sensory decrease	Lesions causing sensory increase
Medial lobule V	(0, -62, −16)	**x**	**x**	**x**
Bilateral lobule V	(±10, −58, −20)	**x**	**x**	**x**
Lobule X	(± 28, −51, −43)		**x**	
Medial lobule VIIIa	(0, −63, −34)	**x**	**x**	**x**
Substantia nigra	(±6, −20, 12)	**x**		**x**
Medial Orbitofrontal cortex	(±14, 56, −14)	**x**		**x**

The sensory network is defined by specific and sensitive connections to Lobule V and medial Lobule VIIIa of the cerebellum, substania nigra, and medial orbitofrontal cortex. The sensory decrease network is defined by specific and sensitive connections to the shared cerebellar regions and Lobule X. The sensory increased network is defined by specific and sensitive connections to Lobule V, medial Lobule VIIIa, substantia nigra, and medial orbitofrontal cortex.

#### Domain-specific connections within sensory sensitivity decrease

**Figure 7:**
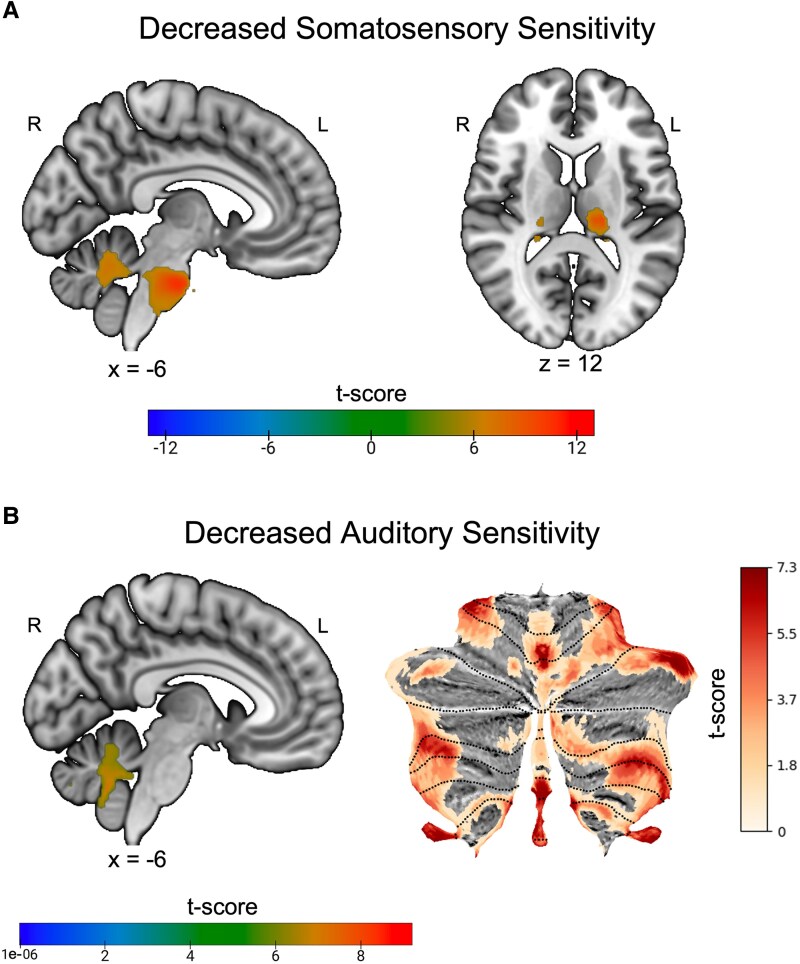
**Lesion network localization of decreased sensory sensitivity by modality. (A)** Functional connectivity specific to lesions causing decreased somatosensory changes (*n* = 15) compared to a large cohort of lesions causing other symptoms (*n* = 659) via two-sample *t*-test (family-wise error (FWE) *P* < 0.05). **(B)** Functional connectivity specific to lesions causing decreased auditory sensory changes (*n* = 13) compared to a large cohort of lesions causing other symptoms via two-sample *t*-test (FWE corrected *P* < 0.05).

Of those with decreased sensory perception, about half of the patients (*n* = 13) experienced auditory changes, while the other patients (*n* = 15) experienced somatosensory changes. Lesions associated with somatosensory decrease displayed sensitive and specific connectivity to the thalamus and pons, while lesions associated with auditory decrease had connectivity to Lobule X (± 28, −51, −43) (Fig. 7A-B).

#### Domain-specific connections independent of direction

Lesions associated with auditory changes displayed connectivity to the temporal lobes and cerebellum, while somatosensory lesions displayed connectivity to S1/M1 and the cerebellum.

## Discussion

In this study, we sought to identify whether lesions that alter somatosensory and auditory stimuli processing, i.e. sensitivity, affect a consistent set of brain networks to understand which higher-level networks are critical to general sensory processing. We found that, independent of the direction of sensitivity change (hypo- versus hyper-) and modality (somatosensory versus auditory), lesions affecting sensory sensitivity intersect a network defined by positive connectivity to Lobule V and medial Lobule VIIIa of the cerebellum, as well as the Substantia Nigra, and negative connectivity to medial orbitofrontal cortex.

### Cerebellum involvement

Across our cohort and sub-analysis networks, we observed consistent cerebellar lesion connectivity, further endorsing its role in general sensory processing and awareness. Cerebellar impairment has been tied to sensory disruptions in primary and higher-order tasks.^27,28^

Notably, medial Lobule V seen here is also part of the functional connectivity-defined SMN,^29^ and shares connections to both motor and somatosensory cortices.^30,31^ While not surprising, the SMN has been implicated in psychomotor disturbances in bipolar disorder^32^ and in the altered adaptive responses/heightened sensitivity to stimuli in Tourette syndrome.^33^

Medial Lobule VIIIa, also seen in our work here, is more consistently identified with the VAN,^29^ with strong connectivity to the prefrontal cortex and has been implicated in executive function deficits. The VAN, activated when attention is reoriented to unexpected cues or between tasks, is directly reliant on bottom-up processing and sensory gating/sensitivity. Our results here may indicate where sensory gating occurs, allowing the ventral attention network to integrate novel stimuli and respond accordingly. Disruption to the VAN has been associated with attentional and sensory disruption in schizophrenia,^34^ ADHD,^35^ and ASD.^36,37^

### Are “Salience’ error signals a cause of altered sensitivity?

One common thread between the lesion-connections noted here, i.e. the cerebellum, thalamus, medial orbitofrontal region, and substantia nigra, is their shared roles in salience and error signalling, as well as sensory gating. Salience dictates that natural, predictable stimuli should be compressed, whereas novel, varied stimuli should hold higher priority and require more attention.^38^ Salience refinement relies on predictive coding that is dependent on error signals generated by the cerebellum. Error signals are generated when there is a mismatch between predicted and actual sensory input through the interaction of climbing fibres and Purkinje spikes.^39^ Higher-order regions provide predictions to lower-order regions where comparisons to inputs are made, and these error signals are fed back to higher-order regions to improve future predictions.^39^

Disruption in error-based learning prevents the improvement of predictions, thus maintaining inaccurate perceptions. Hallucinations are likely a phenomenon involving disproportional weighting of expectations relative to reality.^40^ Our conjunction analysis revealed that lesions associated with auditory and visual hallucinations and sensory changes both localize to the cerebellar vermis, reinforcing that damage to this region decreases sensitivity to errors.^15,41^ This process is particularly worsened under ambiguous sensory input, such as after stroke. Thus, decreased sensory input in post-stroke patients may contribute to greater reliance on prior experiences, resulting in sensitivity changes. Connectivity of the vermis to other regions, such as the frontal cortex, as seen in the sensory and hallucinations cohorts, has also been shown to be modulated with intermittent theta burst stimulation (iTBS) in patients with schizophrenia^42^−a disorder often involving hallucinations–providing converging evidence that this region has clinical significance.

The level of significance placed on incoming stimuli—sensory gating—is thought to occur in the thalamus,^43-45^ which in turn modulates topographically selective and precisely timed cortical neurons for the gating and maintenance of task-relevant information.^46^ Animal studies have demonstrated that thalamocortical neurons have distinct firing patterns that signal open or closed gating based on peripheral or central stimuli.^47,48^ These thalamocortical circuits are modulated by the cerebellum, with stronger thalamocerebellar connectivity correlated with increased sensory sensitivity, specifically in ASD.^49^ The cerebellum also shares connections to the salience network, where Purkinje spike activity reflects the salience of sensory stimuli.^50^ Disruption to salience coding can either allow for all stimuli (even if not novel) to be coded with significance, resulting in increased sensitivity, or overly limit the stimuli (even if novel) to be coded with less significance, resulting in decreased sensitivity.

Another region in our sensitivity network is the bilateral medial orbitofrontal cortex, which plays a critical role in multiple processes, ranging from sensory integration to reward processing.^51^ In particular, the OFC has connections to the secondary somatosensory cortex and primary auditory cortex.^52,53^ Separate from the work here, OFC damage has also been associated with altered somatosensory processing via voxel-based lesion-symptom mapping analyses of stroke lesions with somatosensory deficits^6^ and observations that damage to the OFC can cause reduced auditory sensitivity.^54^ Clinically, OCD patients with increased cross-modal sensitivity show altered OFC connectivity.^55^ These clinical findings and our results are consistent with the theory that medial OFC compares and contrasts the subjective value of potential outcomes, while the lateral OFC, not seen here, computes these potential outcomes, their sensory features, and their subjective value.^56^

Finally, the substantia nigra, also noted in our modality-independent sensitivity network, appears to have a role in salience coding.^57^ As one example, sensory neglect, an extreme case of salience dysfunction, has been reported after lesioning of the substantia nigra in animal studies,^58^ while previous connectivity-based work parcellating the substantia nigra has demonstrated connections to somatomotor regions in humans, consistent with our findings.^57^

### Differential lesion connectivity associated with increased versus decreased sensory sensitivity

We found increased sensitivity was associated with impaired connections to the thalamus and substantia nigra, while decreased sensitivity was associated with impaired connections to Lobule X in the inferior cerebellar vermis.

Increased sensitivity was also associated with lesion connectivity to S1/M1 regions since all the patients had increased somatosensory sensitivity. Lesions that induce neurochemical, excitotoxic, and inflammatory changes can heighten neuronal excitability and promote central sensitization, ultimately resulting in central post-stroke pain.^59^ In contrast, somatosensory decrease was associated with thalamus, who’s role in sensory gating has been discussed above. Of note, a recent voxel-wise lesion-symptom mapping (VLSM) study found a strong association between post-stroke somatosensory deficits and the secondary somatosensory cortex.^6^ They did not find a significant association with the thalamus; however, this may be due to the use of VLSM, which is less sensitive to effects distant in the brain, i.e. diaschisis, or network effects, compared to lesion network mapping. On the other hand, auditory decrease was linked to Lobule X, which is functionally connected to the vestibular system.^60^ The vestibular system is involved in auditory metrical encoding,^61^ reinforcing Lobule X’s plausible role in decreased auditory sensitivity.

### Auditory and somatosensory primary cortex

Lesions associated with auditory hyposensitivity were primarily connected to the temporal lobes, where primary auditory cortices are located. Similarly, somatosensory-altering lesions were connected to the primary motor and somatosensory cortices.

Our findings identify critical neuroanatomy involved in the modulation of sensory input. This has important implications for potential targeted neuromodulatory approaches to recovery after injury or non-lesional developmental disorders. For instance, auditory processing is critical to spoken language and verbal communication, and disruption during development can prevent individuals from observing and acquiring language from the surrounding environment.^62^ Somatosensory processing is critical to understanding both tactile sensitivities and altered pain-related behaviours. Altered somatosensory sensitivity during development can affect individuals’ ability to develop motor skills and non-verbal communication.^63^ Furthermore, disorders of somatosensory sensitivity can alter pain thresholds; diminished processing of pain can interfere with harm avoidance, while heightened processing can lead to sensation-seeking behaviours that are often injurious or maladaptive.

### Limitations

While our findings are illuminating, there are several limitations. These include a small sample size of 61, with 48 experiencing somatosensory changes. Variation across case reports and larger studies in symptom characterization, incomplete lesion tracing via 2D figures, and differences in symptom onset timing could affect the results presented here. However, 2D lesion tracing has been found to be sufficient for LNM.^13,25,64^ Additionally, we have kept our characterization broad to reduce the impact of fine-grained symptom classification while focusing on categorical consistencies. Since we could not obtain functional imaging from the patients, a normative connectome was used to approximate lesion connectivity, which limits the certainty that the networks were actually disrupted in each patient; however, this method has proved to be highly informative across a wide range of studies to date and is more reflective of the networks likely to be impaired by injury.

### Future directions

Future studies that prospectively characterize sensory changes across all sensory modalities would be highly advantageous to determine if our modality-independent findings are indeed independent across all modalities. Assessment of focal structural and functional brain alterations in non-lesional cohorts with a high degree of sensory abnormalities would also provide confirming or opposing converging evidence to the results described here.

## Conclusion

In conclusion, our analysis identified the cerebellum, thalamus, and prefrontal cortex as key regions involved in cross-modality sensory processing—specifically related to subjective sensitivity. These regions and the networks connecting them may represent potential targets for non-invasive neuromodulation techniques, such as transcranial magnetic stimulation (TMS), to normalize altered sensitivity in clinical populations.

## Supplementary Material

fcaf463_Supplementary_Data

## Data Availability

The functional connectivity data used in this study is available online through the Harvard Dataverse at: https://doi.org/10.7910/DVN/ILXIKS , and the pipeline used to prepare the functional connectivity data is available at: https://github.com/bchcohenlab/BIDS_to_CBIG_fMRI_Preproc2016. Sensory Lesion Tracings are available upon reasonable request. Boston Lesion Repository data requests should be directed to the Center for Brain Circuit Therapeutics.
