# Supplementary material for: Network localization of altered auditory and somatosensory sensitivity based on causal brain lesions
Source: Brain Commun. 2025 Nov 24;7(6):fcaf463. doi: 10.1093/braincomms/fcaf463 (PMC12679709; doi:10.1093/braincomms/fcaf463)
Supplement: fcaf463_Supplementary_Data [file fcaf463_supplementary_data.docx]

**Supplementary Table 1**

| Patient # | Reference | Patient no. | Age at Onset | Sex |
| --- | --- | --- | --- | --- |
| 1 | Bowsher 2005 [1] | 1 | Unknown | Unknown |
| 2 |  | 4 | Unknown | Unknown |
| 3 |  | 5 | Unknown | Unknown |
| 4 |  | 6 | Unknown | Unknown |
| 5 | Chen 2016 [2] | 1 | 67 | F |
| 6 |  | 2 | 47 | M |
| 7 |  | 3 | 61 | F |
| 8 |  | 4 | 65 | M |
| 9 |  | 5 | 54 | F |
| 10 |  | 6 | 43 | M |
| 11 |  | 7 | 58 | F |
| 12 |  | 8 | 43 | M |
| 13 | Convers 2020 [3] | 1 | 69 | F |
| 14 |  | 2 | 67 | M |
| 15 |  | 3 | 70 | M |
| 16 |  | 4 | 56 | M |
| 17 |  | 5 | 56 | M |
| 18 | Garcia-Larrea 2010 [4] | 1 | 55 | M |
| 19 |  | 2 | 36 | F |
| 20 |  | 3 | 35 | M |
| 21 |  | 4 | 52 | F |
| 22 |  | 5 | 59 | M |
| 23 | Kim 2017 [5] | 3 | 57 | M |
| 24 |  | 8 | 66 | M |
| 25 |  | 13 | 78 | M |
| 26 |  | 18 | 76 | M |
| 27 | Hanada 2022 [6] |  | 53 | F |
| 28 | He 2022 [7] |  | 47 | F |
| 29 | Ishibashi 2020 [8] |  | 72 | M |
| 30 | Lorenz 1998 [9] |  | 47 | M |
| 31 | Kalita 2011 [10] | 1 | Unknown | Unknown |
| 32 |  | 2 | Unknown | Unknown |
| 33 | Kim 2003 [11] | 1 | 47 | F |
| 34 |  | 2 | 68 | M |
| 35 |  | 3 | 64 | F |
| 36 |  | 4 | 61 | M |
| 37 |  | 5 | 41 | M |
| 38 |  | 6 | 64 | M |
| 39 |  | 7 | 70 | F |
| 40 |  | 8 | 60 | M |
| 41 |  | 9 | 58 | F |
| 42 |  | 10 | 52 | M |
| 43 |  | 11 | 51 | M |
| 44 |  | 17 | 49 | F |
| 45 |  | 19 | 42 | M |
| 46 | Nogami 2022 [12] |  | 57 | M |
| 47 | Pickering 2009 [13] |  | 56 | M |
| 48 | Spitoni 2016 [14] |  | 57 | F |
| 49 | Dietze 2022 [15] | 3 | 72 | Unknown |
| 50 |  | 10 | 73 | Unknown |
| 51 |  | 13 | 76 | Unknown |
| 52 |  | 21 | 66 | Unknown |
| 53 |  | 32 | 75 | Unknown |
| 54 | Ebrahimzadeh 2023 [16] |  | 27 | F |
| 55 | Gwak 2021 [17] |  | 41 | F |
| 56 | Kim 2016 [18] |  | 54 | M |
| 57 | Kinouchi 2021 [19] |  | 73 | F |
| 58 | Lowry 2004 [20] |  | 63 | M |
| 59 | Lu 2024 [21] |  | 44 | M |
| 60 | Kim 2022 [22] |  | 51 | M |
| 61 | Yuan 2023 [23] |  | 44 | M |

Supplementary Table 1: Patients with acquired sensory sensitivity. Age of injury and sex of patients

References:

1. Bowsher D. Allodynia in Relation to Lesion Site in Central Post-Stroke Pain. *J Pain*. 2005;6(11):736-740. doi:10.1016/j.jpain.2005.06.009

2. Chen L, Li C, Zhai J, et al. Altered Resting-State Signals in Patients with Acute Stroke In or Under the Thalamus. *Neurosci Bull*. 2016;32(6):585-590. doi:10.1007/s12264-016-0064-3

3. Convers P, Creac’h C, Beschet A, Laurent B, Garcia‐Larrea L, Peyron R. A hidden mesencephalic variant of central pain. *Eur J Pain*. 2020;24(7):1393-1399. doi:10.1002/ejp.1588

4. Garcia-Larrea L, Perchet C, Creac’h C, et al. Operculo-insular pain (parasylvian pain): a distinct central pain syndrome. *Brain*. 2010;133(9):2528-2539. doi:10.1093/brain/awq220

5. Kim JS, Kang MK, Cho AJ, Seo YB, Kim KI. Complicated infective endocarditis: a case series. *J Med Case Reports*. 2017;11(1):128. doi:10.1186/s13256-017-1274-7

6. Hanada K, Yokoi K, Kashida N, Shimomura R, Murata D, Hirayama K. Midlateral medullary infarction presenting with isolated thermoanaesthesia: a case report. *BMC Neurol*. 2022;22(1):268. doi:10.1186/s12883-022-02796-x

7. He S, Chen Q, Jing Z, Gu L, Luo K. Avellis syndrome with ipsilateral prosopalgia, glossopharyngeal neuralgia, and central post-stroke pain: A case report and literature review. *Medicine (Baltimore)*. 2022;101(39):e30669. doi:10.1097/MD.0000000000030669

8. Ishibashi K, Miura Y, Hirata K, Toyohara J, Ishii K. Relationship between the temporal course of astrogliosis and symptom improvement in cerebral infarction: report of a case monitored using 18F-THK5351 positron emission tomography. *BMC Med Imaging*. 2020;20(1):81. doi:10.1186/s12880-020-00481-4

9. Lorenz J, Kohlhoff H, Hansen HC, Kunze K, Bromm B. Aβ-fiber mediated activation of cingulate cortex as correlate of central post-stroke pain: *NeuroReport*. 1998;9(4):659-663. doi:10.1097/00001756-199803090-00018

10. Kalita J, Kumar B, Misra UK, Pradhan PK. Central Post Stroke Pain: Clinical, MRI, and SPECT Correlation. *Pain Med*. 2011;12(2):282-288. doi:10.1111/j.1526-4637.2010.01046.x

11. Kim JS. Central post-stroke pain or paresthesia in lenticulo-capsular hemorrhages. *Neurology*. 2003;61(5):679-682. doi:10.1212/WNL.61.5.679

12. Nogami C, Hanada K, Yokoi K, et al. A Patient with a Unilateral Insular Lesion Showing Bilaterally Reduced Perception of Noxious Stimulation. *Intern Med*. 2022;61(4):541-546. doi:10.2169/internalmedicine.6878-20

13. Pickering AE, Thornton SR, Love-Jones SJ, Steeds C, Patel NK. Analgesia in conjunction with normalisation of thermal sensation following deep brain stimulation for central post-stroke pain. *Pain*. 2009;147(1):299-304. doi:10.1016/j.pain.2009.09.011

14. Spitoni GF, Pireddu G, Galati G, Sulpizio V, Paolucci S, Pizzamiglio L. Caloric Vestibular Stimulation Reduces Pain and Somatoparaphrenia in a Severe Chronic Central Post-Stroke Pain Patient: A Case Study. Sirigu A, ed. *PLOS ONE*. 2016;11(3):e0151213. doi:10.1371/journal.pone.0151213

15. Dietze A, Sörös P, Bröer M, et al. Effects of acute ischemic stroke on binaural perception. *Front Neurosci*. 2022;16:1022354. doi:10.3389/fnins.2022.1022354

16. Ebrahimzadeh K, Tavassol HH, Mousavinejad SA, et al. The Sensorineural Hearing Loss Related to a Rare Infratentorial Developmental Venous Angioma: A Case Report and Review of Literature. *J Neurol Surg Part Cent Eur Neurosurg*. 2023;84(03):288-294. doi:10.1055/s-0041-1725960

17. Gwak DW, Park E, Park JS, et al. Alterations of functional connectivity in auditory and sensorimotor neural networks: A case report in a patient with cortical deafness after bilateral putaminal hemorrhagic stroke. *Medicine (Baltimore)*. 2021;100(3):e24302. doi:10.1097/MD.0000000000024302

18. Kim JH, Roh KJ, Suh SH, Lee KY. Republished: Improvement of sudden bilateral hearing loss after vertebral artery stenting. *J NeuroInterventional Surg*. 2016;8(3):e12-e12. doi:10.1136/neurintsurg-2014-011595.rep

19. Kinouchi T, Ishitani K, Uyama S, Miyamoto T, Fujimoto N, Ueta H. Basilar artery occlusion presenting as sudden bilateral deafness: a case report. *J Med Case Reports*. 2021;15(1):111. doi:10.1186/s13256-020-02574-8

20. Lowry LD, Eisenman LM, Saunders JC. An Absence of Tinnitus: *Otol Neurotol*. 2004;25(4):474-478. doi:10.1097/00129492-200407000-00013

21. Lu RY, Zhu HK, Wang S, Zhang YX. Bilateral hearing loss as the initial presentation of reversible Wernicke’s encephalopathy with splenial lesion. *Neurol Sci*. 2024;45(9):4593-4596. doi:10.1007/s10072-024-07546-0

22. Kim YS, Tehrani ASS, Lee HS. Sudden unilateral hearing loss and vertigo following isolated cerebellar hypoperfusion without infarction due to vertebral artery dissection. *BMC Neurol*. 2022;22(1):489. doi:10.1186/s12883-022-03024-2

23. Yuan Z, Xiang L, Liu R, Yue W. Case report: Bilateral sudden deafness in acute middle cerebellar peduncle infarction: central or peripheral? *Front Med*. 2023;10:1174512. doi:10.3389/fmed.2023.1174512

**Supplementary Table 2**

| Symptom | Number subjects | Citation | Doi |
| --- | --- | --- | --- |
| Akinetic mutism | 28 | Darby et al., 2018 [1] | 10.1073/pnas.1814117115 |
| Alien limb | 53 | Darby et al., 2018 [1] | 10.1073/pnas.1814117115 |
| Amnesia | 53 | Ferguson et al., 2019 [2] | 10.1038/s41467-019-11353-z |
| Anton syndrome | 23 | Kletenik et al. 2023 [3] | 10.1002/ana.26709 |
| Aphasia | 12 | Boes et al., 2015 [4] | 10.1093/brain/awv228 |
| Asterixis | 30 | Joutsa et al., 2018 [5] | 10.1002/ana.25285 |
| Blindsight | 34 | Kletenik et al., 2021 [6] | 10.1002/ana.26292 |
| Coma | 12 | Fischer et al., 2016 [7] | 10.1212/WNL.0000000000003404 |
| Confabulation | 25 | Bateman et al., 2023 [8] | 10.1176/appi.neuropsych.20220160 |
| Cortical blindness | 35 | Kletenik et al., 2021 [6] | 10.1002/ana.26292 |
| Delusion | 32 | Darby et al., 2017 [9] | 10.1093/brain/aww288 |
| Cervical dystonia | 25 | Corp et al., 2019 [10] | 10.1093/brain/awz112 |
| Freezing of gait | 14 | Fasano et al., 2017 [11] | 10.1002/ana.24845 |
| Hallucination | 89 | Boes et al. 2015 Brain, [4]  Kim et al. 2021 Mol Psych [12] | 10.1093/brain/awv228,  10.1038/s41380-019-0565-3 |
| Hemichorea | 29 | Laganiere et al., 2016 [13] | 10.1212/WNL.0000000000002741 |
| Holmes tremor | 36 | Joutsa et al., 2019 [14] | 10.1002/ana.25618 |
| Loss of consciousness | 16 | Snider et al., 2020 [15] | 10.1002/hbm.24892 |
| Central poststroke pain | 23 | Boes et al., 2015 [4] | 10.1093/brain/awv228 |
| Parkinsonism | 29 | Joutsa et al., 2018 [16] | 10.1093/brain/awy161 |
| Prosopagnosia | 44 | Cohen et al., 2019 [17] | 10.1093/brain/awz332 |

Supplementary Table 2: Control Lesions from the Boston Lesion Repository. The specific symptoms included in the control dataset of 659 lesions, as well as the number of lesions for each symptom.

References:

1. R. R. Darby, J. Joutsa, M. J. Burke, and M. D. Fox, “Lesion network localization of free will,” PNAS, vol. 115, no. 42, pp. 10792–10797, Oct. 2018, doi: 10.1073/pnas.1814117115.
2. M. A. Ferguson et al., “A human memory circuit derived from brain lesions causing amnesia,” Nature communications, vol. 10, no. 1, p. 3497, Aug. 2019, doi: 10.1038/s41467-019-11353-z.
3. I. Kletenik, K. Gaudet, S. Prasad, A. L. Cohen, and M. D. Fox, “Network Localization of Awareness in Visual and Motor Anosognosia,” Ann Neurol, vol. 94, no. 3, pp. 434–441, Sep. 2023, doi: 10.1002/ana.26709.
4. A. D. Boes et al., “Network localization of neurological symptoms from focal brain lesions,” Brain, vol. 138, no. Pt 10, pp. 3061–3075, Oct. 2015, doi: 10.1093/brain/awv228.
5. J. Joutsa et al., “Identifying therapeutic targets from spontaneous beneficial brain lesions,” Ann Neurol, vol. 84, no. 1, pp. 153–157, Jul. 2018, doi: 10.1002/ana.25285.
6. I. Kletenik et al., “Network Localization of Unconscious Visual Perception in Blindsight,” Ann Neurol, Dec. 2021, doi: 10.1002/ana.26292.
7. D. B. Fischer et al., “A human brain network derived from coma-causing brainstem lesions,” Neurology, vol. 87, no. 23, pp. 2427–2434, 2016, doi: 10.1212/WNL.0000000000003404.
8. J. R. Bateman et al., “Network Localization of Spontaneous Confabulation,” The journal of neuropsychiatry and clinical neurosciences, pp. appineuropsych20220160–appineuropsych20220160, 2023, doi: 10.1176/appi.neuropsych.20220160.
9. R. R. Darby, S. Laganiere, A. Pascual-Leone, S. Prasad, and M. D. Fox, “Finding the imposter: brain connectivity of lesions causing delusional misidentifications,” Brain: a journal of neurology, vol. 140, no. 2, pp. 497–507, Feb. 2017, doi: 10.1093/brain/aww288.
10. D. T. Corp et al., “Network localization of cervical dystonia based on causal brain lesions,” Brain, vol. 142, no. 6, pp. 1660–1674, Jun. 2019, doi: 10.1093/brain/awz112.
11. A. Fasano, S. E. Laganiere, S. Lam, and M. D. Fox, “Lesions causing freezing of gait localize to a cerebellar functional network,” Ann Neurol, vol. 81, no. 1, pp. 129–141, Jan. 2017, doi: 10.1002/ana.24845.
12. N. Y. Kim et al., “Lesions causing hallucinations localize to one common brain network,” Molecular psychiatry, vol. 26, no. 4, pp. 1299–1309, Apr. 2021, doi: 10.1038/s41380-019-0565-3.
13. S. Laganiere, A. D. Boes, and M. D. Fox, “Network localization of hemichorea-hemiballismus,” Neurology, vol. 86, no. 23, pp. 2187–2195, Jun. 2016, doi: 10.1212/WNL.0000000000002741.
14. J. Joutsa, L. C. Shih, and M. D. Fox, “Mapping holmes tremor circuit using the human brain connectome,” Ann Neurol, vol. 86, no. 6, pp. 812–820, Dec. 2019, doi: 10.1002/ana.25618.
15. S. B. Snider et al., “Cortical lesions causing loss of consciousness are anticorrelated with the dorsal brainstem,” Human Brain Mapping, vol. 41, no. 6, pp. 1520–1531, Apr. 2020, doi: 10.1002/hbm.24892.
16. J. Joutsa, A. Horn, J. Hsu, and M. D. Fox, “Localizing parkinsonism based on focal brain lesions,” Brain, vol. 141, no. 8, pp. 2445–2456, Aug. 2018, doi: 10.1093/brain/awy161.
17. A. L. Cohen, L. Soussand, S. L. Corrow, O. Martinaud, J. J. S. Barton, and M. D. Fox, “Looking beyond the face area: lesion network mapping of prosopagnosia,” Brain, vol. 142, no. 12, pp. 3975–3990, Dec. 2019, doi: 10.1093/brain/awz332.

**Supplementary Figure 1**

| 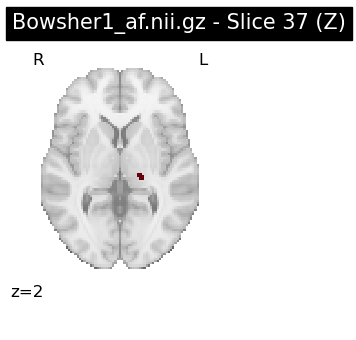 Patient 1 | 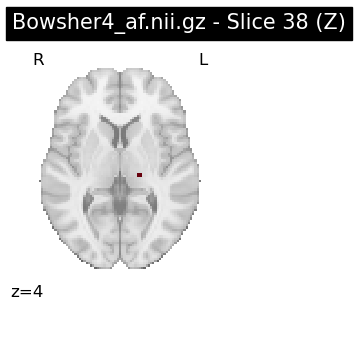 Patient 2 | 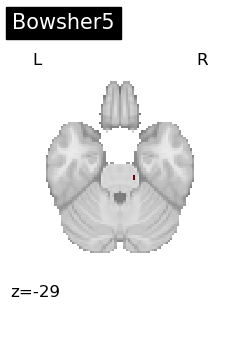 Patient 3 | 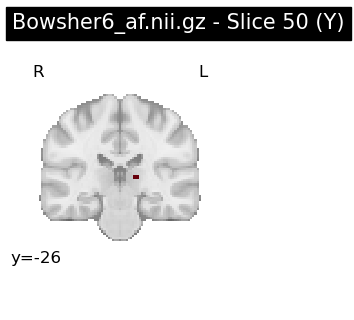 Patient 4 | 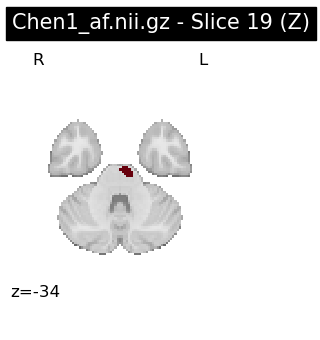 Patient 5 | 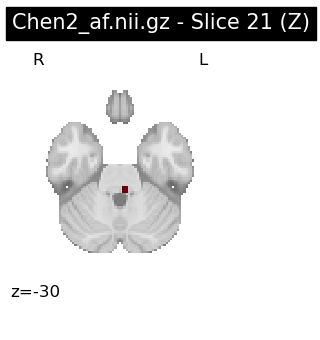 Patient 6 |
| --- | --- | --- | --- | --- | --- |
| 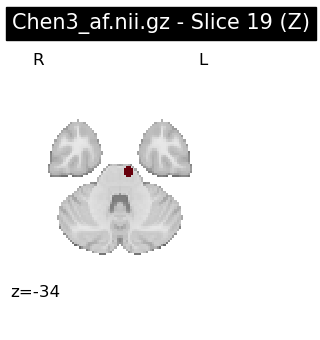 Patient 7 | 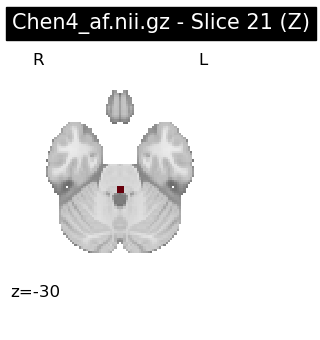 Patient 8 | 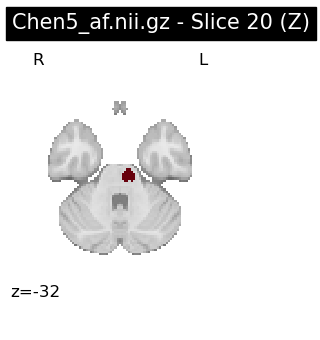 Patient 9 | 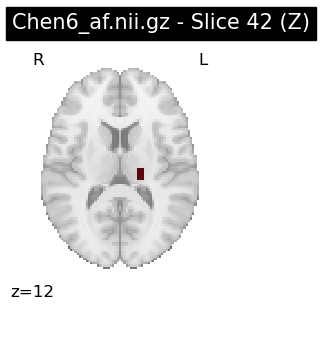 Patient 10 | 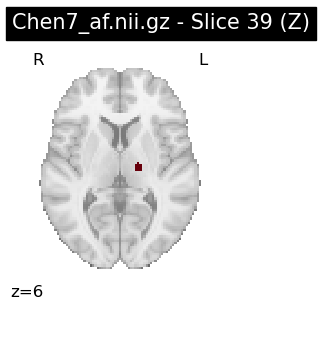 Patient 11 | 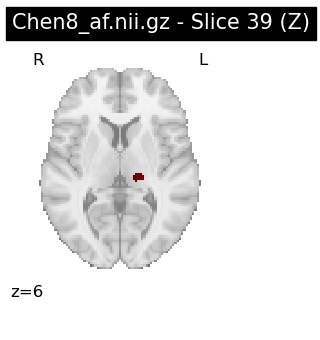 Patient 12 |
| 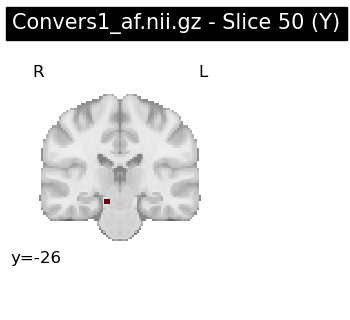 Patient 13 | 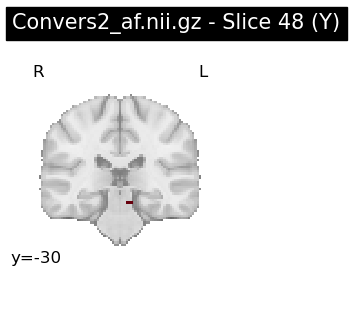 Patient 14 | 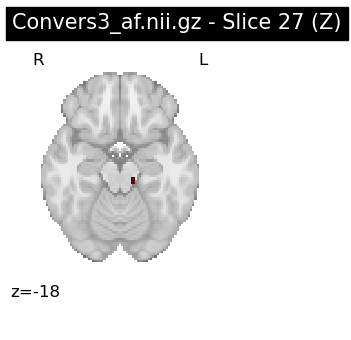 Patient 15 | 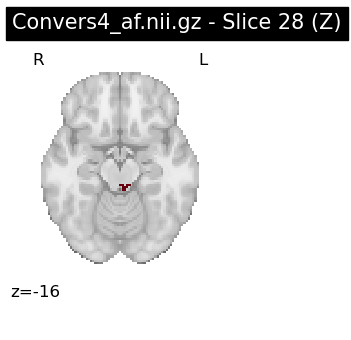 Patient 16 | 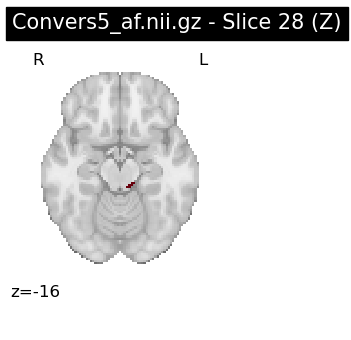 Patient 17 | 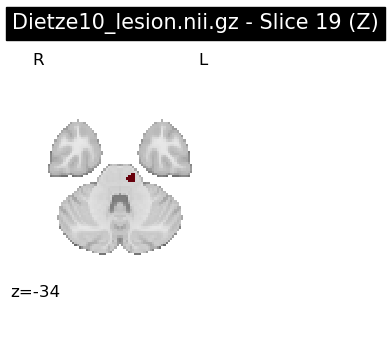 Patient 18 |
| 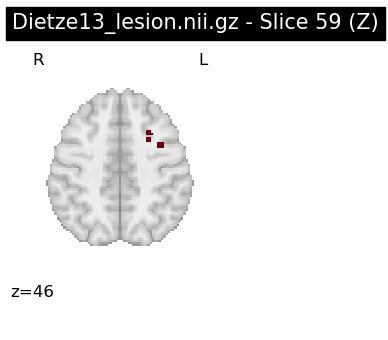 Patient 19 | 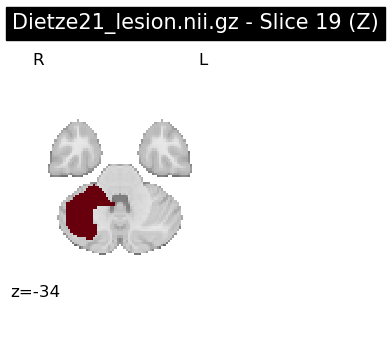 Patient 20 | 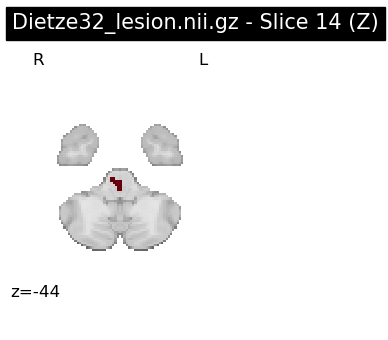 Patient 21 | 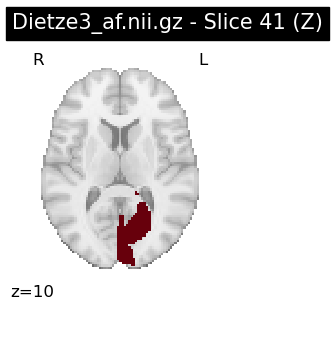 Patient 22 | 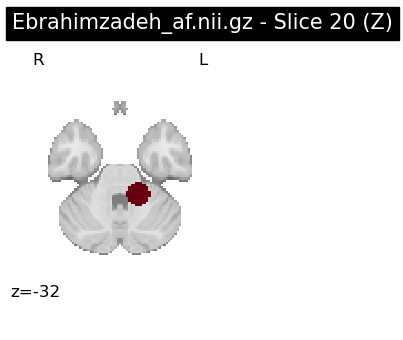 Patient 23 | 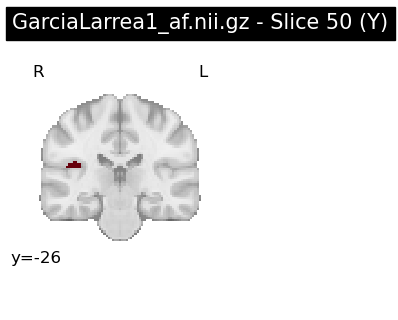 Patient 24 |
| 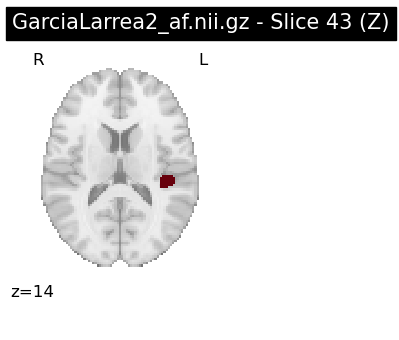 Patient 25 | 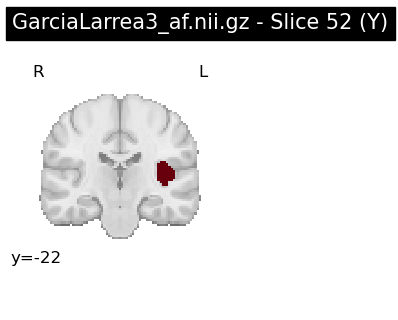 Patient 26 | 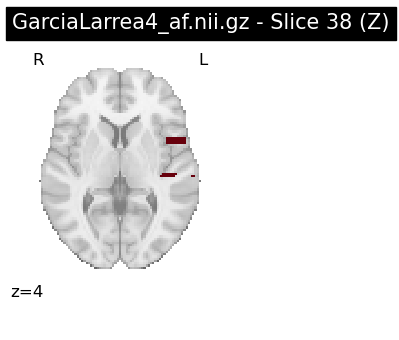 Patient 27 | 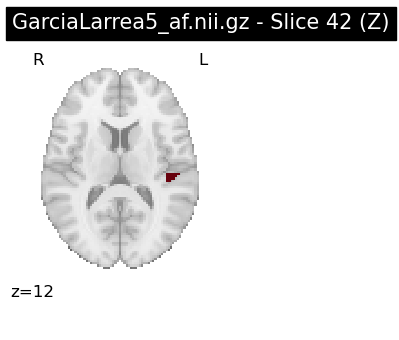 Patient 28 | 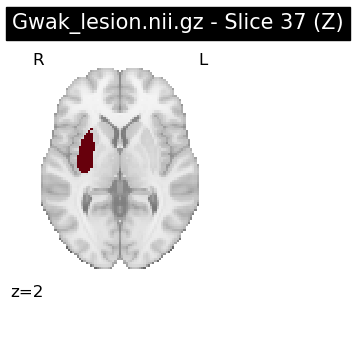 Patient 29 | 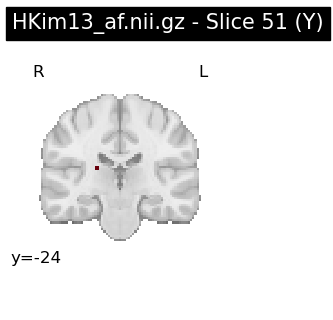 Patient 30 |
| 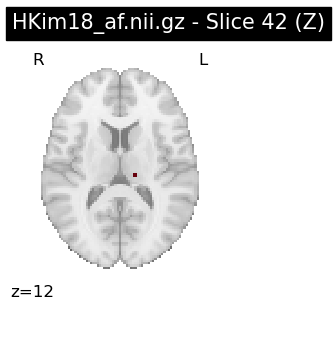 Patient 31 | 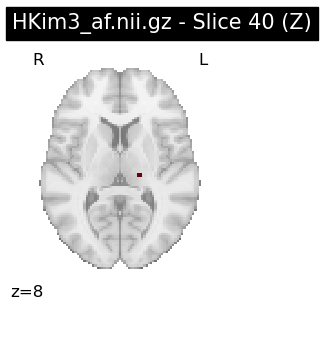 Patient 32 | 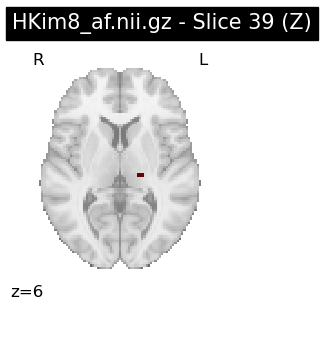 Patient 33 | 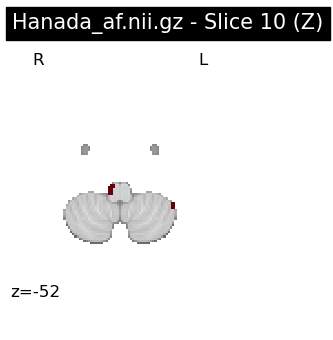 Patient 34 | 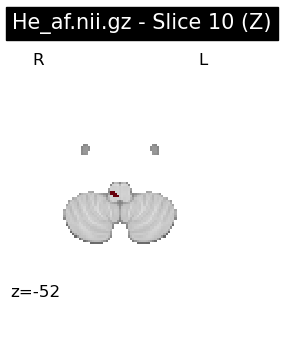 Patient 35 | 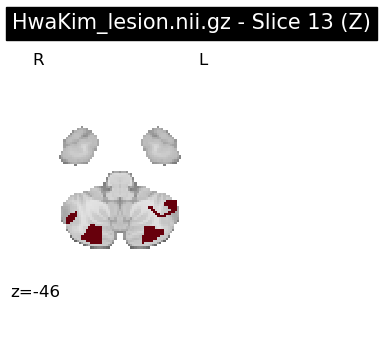 Patient 36 |
| 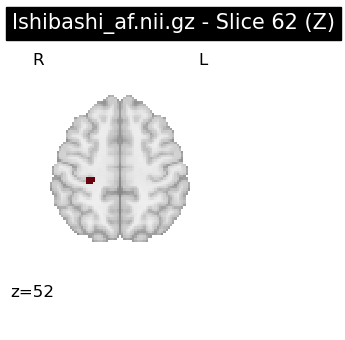 Patient 37 | 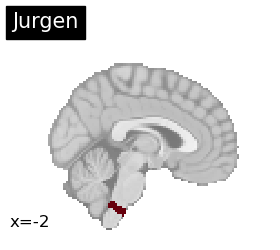 Patient 38 | 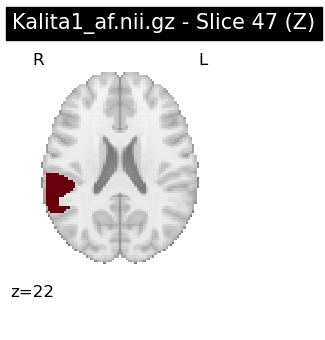 Patient 39 | 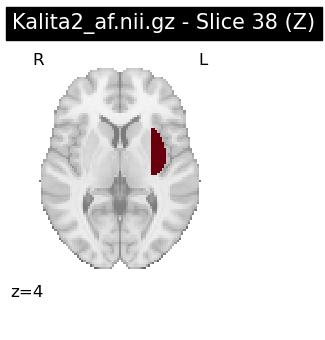 Patient 40 | 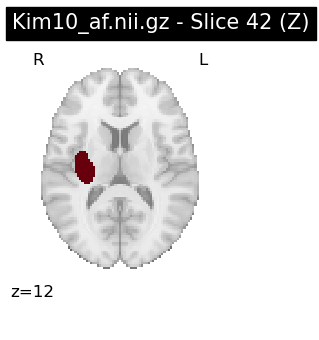 Patient 41 | 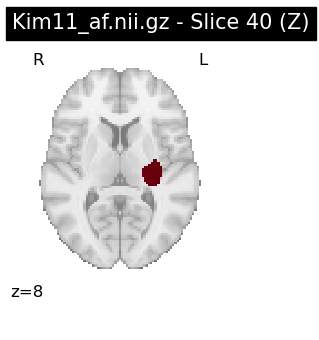 Patient 42 |
| 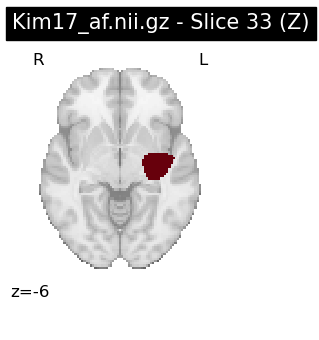 Patient 43 | 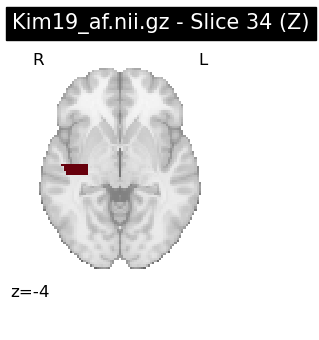 Patient 44 | 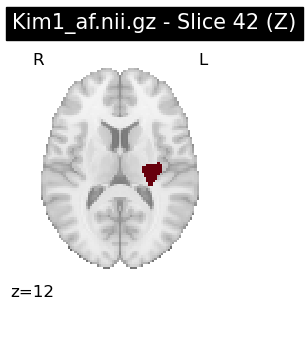 Patient 45 | 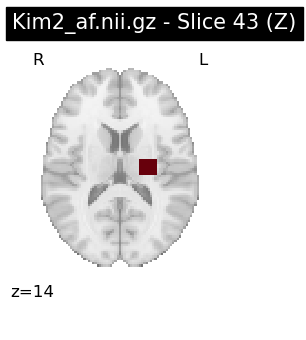 Patient 46 | 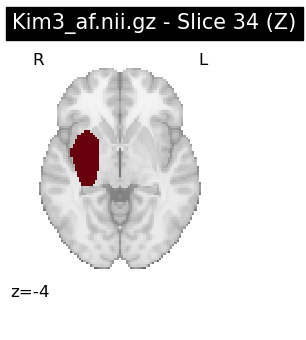 Patient 47 | 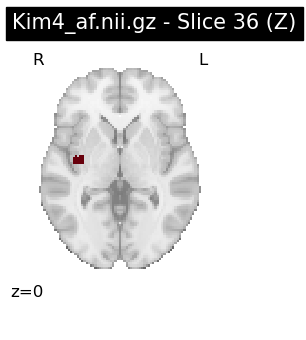 Patient 48 |
| 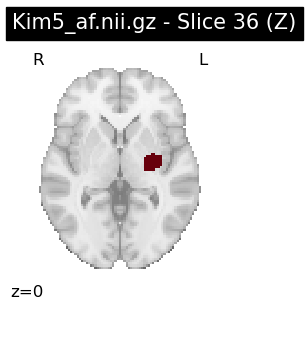 Patient 49 | 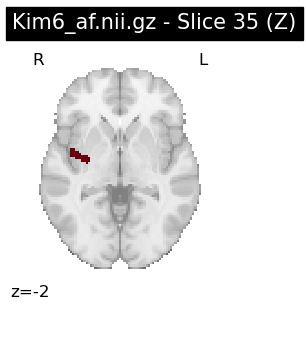 Patient 50 | 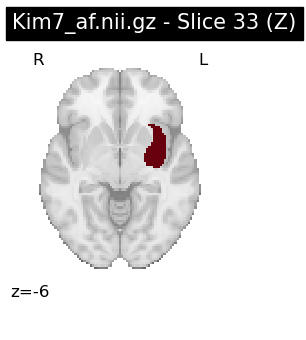 Patient 51 | 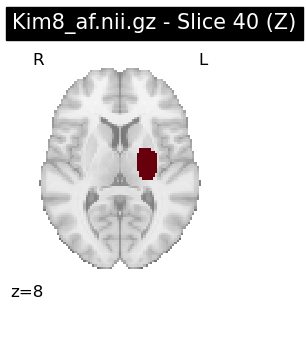 Patient 52 | 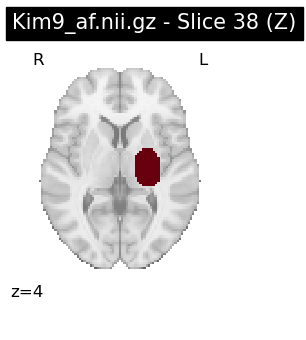 Patient 53 | 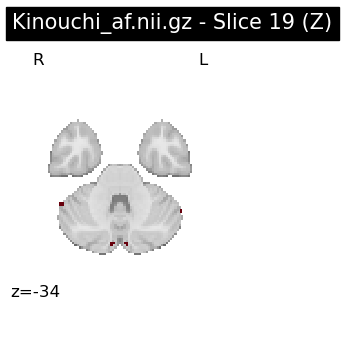 Patient 54 |
| 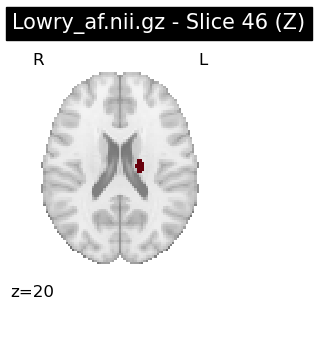 Patient 55 | 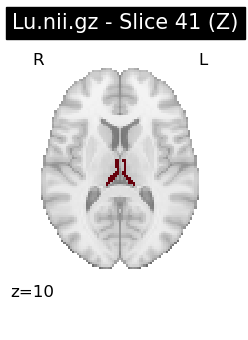 Patient 56 | 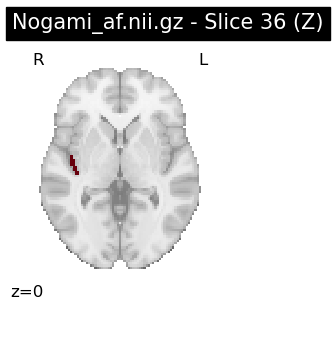 Patient 57 | 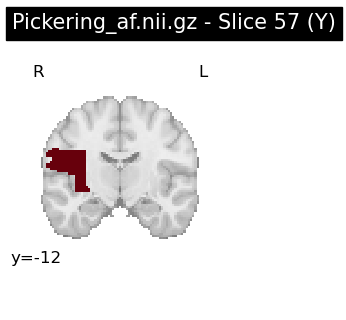 Patient 58 | 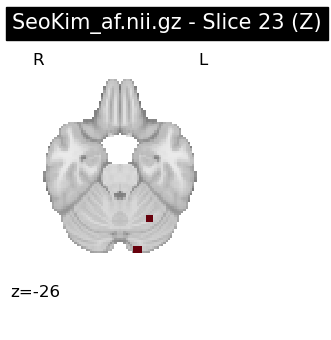 Patient 59 | 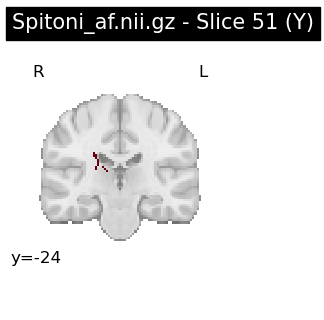 Patient 60 |
| 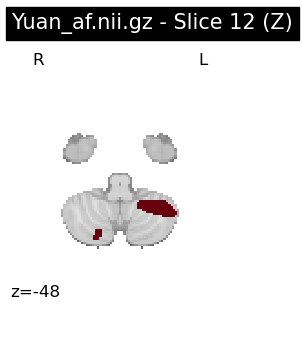 Patient 61 |  |  |  |  |  |

Supplementary Figure 1. Lesions Associated with Somatosensory or Auditory Sensitivity. Representative slices of the 61 lesions associated with somatosensory or auditory sensitivity.
